# Evaluation of the Safety and Efficacy of Coronary Intravascular Lithotripsy for Treatment of Severely Calcified Coronary Stenoses: Evidence From the Serial Disrupt CAD Trials

**DOI:** 10.3389/fcvm.2021.724481

**Published:** 2021-08-19

**Authors:** Bo Liang, Ning Gu

**Affiliations:** ^1^Nanjing University of Chinese Medicine, Nanjing, China; ^2^Nanjing Hospital of Chinese Medicine Affiliated to Nanjing University of Chinese Medicine, Nanjing, China

**Keywords:** coronary intravascular lithotripsy, severely calcified coronary stenoses, optical coherence tomography, Disrupt CAD, cardiology (clinical)

## Abstract

**Background:** Previous understanding holds that rotational atherectomy and modified balloons remain the default strategy for severely calcified coronary stenoses. In recent years, coronary intravascular lithotripsy (IVL) provides new ideas. This study was conducted to evaluate the safety and efficacy of IVL for the treatment of severely calcified coronary stenoses.

**Methods:** The serial Disrupt CAD trials (Disrupt CAD I, Disrupt CAD II, Disrupt CAD III, and Disrupt CAD IV) were included in this study. The safety endpoint was freedom from major adverse cardiovascular events (MACE) in hospital, at 30 days, and at 6 months following the index procedure. The efficacy endpoints included procedural success and angiographic success. Optical coherence tomography (OCT) was used to evaluate the mechanism of action of IVL quantifying the coronary artery calcification (CAC) characteristics and calcium plaque fracture.

**Results:** We enrolled a total of 628 patients with a mean age of 71.8 years, 77.1% males. In these patients, the left anterior descending artery and right coronary artery were the most vulnerable vessels. The diameter stenosis was 64.6 ± 11.6% and the lesion length was 24.2 ± 11.4 mm. IVL had a favorable efficacy (93.0% procedural success, 97.5% angiographic success, and 100.0% stent delivery). Among the 628 patients, 568, 568, and 60 reported MACE endpoints in hospital, at 30 days, and at 6 months, respectively. The results showed that 528, 514, and 55 patients were free from MACE in hospital, at 30 days, and at 6 months, respectively. OCT measurements demonstrated that calcium fracture was the underlying mechanism of action for coronary IVL.

**Conclusions:** IVL is safe and efficient for severely calcified coronary stenoses, and, importantly, calcium fracture facilitated increased vessel compliance and favorable stent expansion.

## Background

In the process of coronary atherosclerosis, coronary artery calcification (CAC) is often accompanied. Even though CAC might have no specific clinical manifestation, this asymptomatic phenomenon is highly prevalent in patients with severe coronary artery disease (CAD) and is associated with major adverse cardiovascular events (MACE) (1). With the help of computed tomography coronary angiography (CTCA), intravascular ultrasound (IVUS), and optical coherence tomography (OCT), the detection of CAC has been greatly improved, but severe CAC still significantly increases the difficulty of percutaneous coronary intervention (PCI) (1, 2). The treatment of calcified lesions includes modified (scoring or cutting) balloons and the application of rotational atherectomy (RA) (3). RA, a complex method utilizing a diamond-tipped burr, can effectively fracture the calcium plaque through the high-speed rotation of its burr and then selectively act on the calcified tissue, resulting in the debulking of calcium plaques (4). RA is a technically demanding procedure reliant on operator experience, and the safety of RA has improved with accumulated experience and maturation of the technique (4, 5). The randomized ROTAXUS (Rotational Atherectomy Prior to TAXUS Stent Treatment for Complex Native Coronary Artery Disease) (3) and PREPARE-CALC (Comparison of Strategies to Prepare Severely Calcified Coronary Lesions) (6) trials implied that RA seems to be successful and is not associated with excessive late lumen loss. However, angiographic and clinical complications of RA are persistent (7) and emphasize the potential for further progress in technology and technique (8, 9). How to treat severely calcified coronary stenoses succinctly and effectively is still a great challenge facing interventional therapy. Coronary intravascular lithotripsy (IVL), based on an established treatment strategy for the renal calculi to disrupt vascular calcium, facilitates the PCI of severely calcified coronary stenoses by using high-pressure ultrasonic energy. IVL is the newest adjunctive method for calcium modification, and recently it is being applied in the clinic and generates promise and encouraging results as its users gain more experience and it becomes readily available worldwide (10). Since the sample sizes of the previously published studies involving IVL for severely calcified coronary stenoses were generally small, we here integrate the serial Disrupt CAD trials (11–14) to further evaluate the safety and efficacy of IVL for the treatment of severely calcified coronary stenoses.

## Methods

The data were extracted from the serial Disrupt CAD trials by two reviewers independently and were crossed checked. All data indicators from these trials can be used for subsequent analysis. After detecting the skewness (15), we estimated the sample mean and standard deviation from the sample size, median, and interquartile range according to previous studies (16, 17), and then we pooled the corresponding data. All the results were descriptive analysis, as described previously (18).

The safety endpoint was freedom from MACE in hospital, at 30 days, and at 6 months following the index procedure. The efficacy endpoints included procedural success and angiographic success (19). MACE composited the occurrence of cardiac death, myocardial infarction (MI), or target vessel revascularization (TVR) (20). MI was defined as a creatine kinase MB isoform (CK-MB) level more than three times the upper limit of lab normal value with or without new pathologic Q waves at discharge (periprocedural MI) and using the Fourth Universal Definition of Myocardial Infarction beyond discharge (spontaneous MI). TVR was revascularization at the target vessel (inclusive of the target lesion) after the completion of the index procedure. Procedural success was defined as the ability of IVL to produce residual diameter stenosis <50% after stenting with no evidence of in-hospital MACE (21). The diameter stenosis was calculated by the fractional flow reserve or the quantitative flow ratio (22–24). Angiographic success was defined as success in facilitating stent delivery with less than 50% residual stenosis and without serious angiographic complications [severe dissection impairing flow (types D–F), perforation, abrupt closure, persistent slow flow, or no reflow] (19). Coronary artery dissections were categorized using the National Heart Lung and Blood Institute classification (25–27).

The OCT guide for calcified coronary lesions resulted in a larger percent stent expansion compared to the IVUS guide (28). In this study, OCT was used to evaluate the mechanism of action of IVL quantifying the CAC characteristics and calcium plaque fracture. Calcium plaque fracture is considered as a new rupture and/or discontinuity of the calcium plaque found on OCT after IVL or stent implantation (29). In order to determine the number of fractures in each lesion, the continuity of calcium plaque fracture was tracked in the whole lesion frame by frame and cross-examined with longitudinal OCT images (12, 30). OCT imaging was planned at three time points (pre-IVL, post-IVL, and following stent deployment at the end of the procedure) to more accurately characterize the extent of calcification and provide insights into the mechanism of IVL in facilitating stent expansion.

Since all the Disrupt CAD trials were single-arm studies, we cannot obtain anything about a control. Considering that RA is widely used for lesion preparation before stent implantation, as confirmed by the European Association of Percutaneous Cardiovascular Interventions (5), we also included two high-quality studies on RA [ROTAXUS (3) and PREPARE-CALC (6), which were performed at three and at two high-volume, experienced interventional study sites in Germany, respectively]. Similarly, the data were extracted by two reviewers independently and pooled as described above.

We pooled Disrupt CAD I (11), Disrupt CAD II (12), Disrupt CAD III (13), and Disrupt CAD IV (14) to the Disrupt CAD group and pooled ROTAXUS (3) and PREPARE-CALC (6) to the RA group. We only selected the same data in the two groups for comparison. The continuous variables, in accordance with the normality distribution, are represented as the mean and standard deviation; otherwise, they are represented as the median and interquartile range. The categorical variables are presented as counts and proportions. All the results were descriptive analysis, as described previously (18).

## Results

### Study Characteristics

Four serial Disrupt CAD trials [Disrupt CAD I (11), Disrupt CAD II (12), Disrupt CAD III (13), and Disrupt CAD IV (14)] were included in our study. All of them were prospective, multicenter, single-arm studies and were conducted in multiple hospitals. Except for the Disrupt CAD IV, the others were all multi-country studies. The inclusion and exclusion criteria for the four studies are listed in Table 1.

**Table 1:** Summary of the serial Disrupt CAD trials.

	**Disrupt CAD I**	**Disrupt CAD II**	**Disrupt CAD III**	**Disrupt CAD IV**
NCT no.	NCT02650128	NCT03328949	NCT03595176	NCT04151628
Design	Prospective multicenter single-arm study conducted at seven hospitals in five countries	Prospective multicenter single-arm study conducted at 15 hospitals in nine countries	Prospective multicenter single-arm study conducted at 47 hospitals in four countries	Prospective multicenter single-arm study conducted at eight hospitals in Japan
Inclusion criteria	Patients with a clinical indication for coronary intervention were required to have one or more lesions requiring percutaneous coronary intervention with a diameter stenosis ≥50%, native coronary artery lesion length ≤ 32 mm, and heavy calcification.	Patients had silent ischemia, unstable or stable angina with evidence of myocardial ischemia, or stabilized acute coronary syndrome without elevation in the cardiac biomarkers. Participants were required to have a single target lesion requiring percutaneous coronary intervention with a diameter stenosis ≥50%, lesion length ≤ 32 mm in native coronary arteries, and severe calcification.	Patients presenting with stable, unstable, or silent ischemia and severely calcified *de novo* coronary artery lesions undergoing percutaneous coronary intervention were eligible for enrollment. Target lesions were ≤ 40 mm in length with reference vessel diameters of 2.5–4.0 mm.	Eligible patients were scheduled for percutaneous coronary intervention and presented with stable, unstable, or silent ischemia and severely calcified *de novo* coronary artery lesions. Target lesions were ≤ 40 mm in length and the target vessel reference diameter ranged from 2.5 to 4.0 mm.
Exclusion criteria	Not reported.	Participants were excluded if there was planned use of atherectomy, specialty balloons, or investigational coronary devices.	Patients with acute myocardial infarction and specific complex lesion features were excluded.	Patients with New York Heart Association class III or IV heart failure, renal failure, or recent myocardial infarction, stroke, or transient ischemic attack were excluded.

The studies included a total of 628 patients with a clinical indication for coronary intervention. The sample size in the trials ranged from 60 patients in the Disrupt CAD I to 384 patients in the Disrupt CAD III. The mean/median age of participants ranged from 70 to 75 years, with the Disrupt CAD IV trial including older patients. Males accounted for the majority of each study (range, 75.0–80.0%). The majority of the population included in our study had hypertension and hyperlipidemia; nearly half had diabetes and previous myocardial infarction; and a small number had previous coronary artery bypass graft, stroke or transient ischemic attack, history of smoking, and renal insufficiency (defined as an estimated glomerular filtration rate <60 ml/min per 1.73 m^2^). According to the angina classification of the Canadian Cardiovascular Society, most patients were in classes I and II (Table 2).

**Table 2:** Baseline and clinical demographics.

	**Disrupt CAD I (*N* = 60)**	**Disrupt CAD II (*N* = 120)**	**Disrupt CAD III (*N* = 384)**	**Disrupt CAD IV (*N* = 64)**	**Total (*N* = 628)**
**Demographics**
Age (years)	72 (66–79)	72.1 ± 9.8	71.2 ± 8.6	75.0 ± 8.0	71.8 ± 9.0
Male	48 (80.0%)	94 (78.3%)	294 (76.6%)	48 (75.0%)	484 (77.1%)
**Medical/surgical history**
Diabetes	18 (30.0%)	38 (31.7%)	154 (40.1%)	31 (48.4%)	241 (38.4%)
Hypertension	48 (80.0%)	96 (80.0%)	342 (89.1%)	53 (82.8%)	539 (85.8%)
Hyperlipidemia	48 (80.0%)	86 (71.7%)	342 (89.1%)	55 (85.9%)	531 (84.6%)
Prior myocardial infarction	24 (40.0%)	31 (25.8%)	69 (18.0%)	13 (20.4%)	137 (21.8%)
Prior coronary artery bypass graft	14 (23.3%)	8 (6.7%)	36 (9.4%)	2 (3.1%)	60 (9.6%)
Prior stroke or transient ischemic attack	8 (13.3%)	4 (3.3%)	29 (7.6%)	13 (20.3%)	54 (8.6%)
History of tobacco use	9 (15.0%)	16 (13.3%)	47 (12.2%)	40 (62.5%)	112 (17.8%)
Renal insufficiency^a^	6 (10.0%)	10 (8.3%)	101 (26.3%)	15 (23.4%)	132 (21.0%)
**Canadian Cardiovascular Society angina classification**
Class 0	0 (0%)	24 (20.0%)	48 (12.5%)	17 (26.6%)	89 (14.2%)
Class I	19 (31.7%)	42 (35.0%)	56 (14.6%)	25 (39.1%)	142 (22.6%)
Class II	29 (48.3%)	36 (30.0%)	142 (37.0%)	21 (32.8%)	228 (36.3%)
Class III	10 (16.7%)	6 (5.0%)	126 (32.8%)	1 (1.6%)	143 (22.8%)
Class IV	2 (3.3%)	2 (1.7%)	9 (2.3%)	0 (0%)	13 (2.1%)
Not assessed	0 (0%)	10 (8.3%)	3 (0.8%)	0 (0%)	13 (2.1%)

*Values are n (%), or median with interquartile range (25–75%), or mean ± standard deviation.*

a *Renal insufficiency was defined as an estimated glomerular filtration rate <60 ml/min per 1.73 m^2^*.

### Lesion Characteristics

For lesion characteristics, the most frequently involved target vessel was the left anterior descending artery, followed by the circumflex artery. The reference vessel diameter was about 3 mm and the minimum lumen diameter was about 1 mm (Disrupt CAD IV did not report the minimum lumen diameter). The incidence of diameter stenosis was more than 60.0% in all trials. The lesion length and the calcified length of the Disrupt CAD III and Disrupt CAD IV were longer than those of the Disrupt CAD I and Disrupt CAD II. Except for 94.2% of severe calcification in the Disrupt CAD II, all other studies achieved 100%. In all studies, the side branch involvement rate was about 30% (Table 3).

**Table 3:** Lesion characteristics.

	**Disrupt CAD I**	**Disrupt CAD II**	**Disrupt CAD III**	**Disrupt CAD IV**	**Total**
Target vessel					
Protected left main artery	1 (1.7%)	1 (0.8%)	6 (1.6%)	1 (1.6%)	9 (1.4%)
Left anterior descending artery	28 (46.7%)	75 (62.5%)	217 (56.5%)	48 (75.0%)	368 (58.6%)
Circumflex artery	8 (13.3%)	14 (11.7%)	49 (12.8%)	4 (6.3%)	75 (11.9%)
Right coronary artery	23 (38.3%)	30 (25.0%)	112 (29.2%)	11 (17.2%)	176 (28.0%)
Reference vessel diameter (mm)	3.0 (2.6–3.2)	3.0 ± 0.5	3.0 ± 0.5	2.9 ± 0.4	3.0 ± 0.5
Minimum lumen diameter (mm)	0.9 (0.6–1.1)	1.2 ± 0.4	1.1 ± 0.4	NA	1.1 ± 0.4 [564]
Diameter stenosis (%)	73 (59–77)	60.0 ± 12.0	65.1 ± 10.8	65.8 ± 10.9	64.6 ± 11.6
Lesion length (mm)	18 (14–25)	19.5 ± 9.8	26.0 ± 11.7	27.5 ± 10.4	24.2 ± 11.4
Calcified length (mm)	21 (12–25)	25.7 ± 12.4	47.9 ± 18.8	49.8 ± 15.5	41.1 ± 20.1
Severe calcification	60 (100%)	113 (94.2%)	384 (100%)	64 (100%)	621 (98.9%)
Lesion assessment					
Concentric	47 (78.3%)	86 (71.7%)	NA	NA	133 (73.9%) [180]
Eccentric	13 (21.7%)	34 (28.3%)	NA	NA	47 (26.1%) [180]
Side branch involvement	17 (28.3%)	36 (30.0%)	115 (29.9%)	22 (34.4%)	190 (30.3%)

*Values are n (%) [N], or median with interquartile range (25–75%), or mean ± standard deviation. NA, not applicable*.

### Procedural Details

Regarding the total procedure time, the Disrupt CAD I took the longest, up to an hour and a half, and the rest took about an hour. Similarly, the Disrupt CAD I also had a longer fluoroscopy time than did others. The Disrupt CAD II and the Disrupt CAD III reported contrast volumes of 181.9 ± 66.4 and 167.9 ± 71.9 ml, respectively. Only the Disrupt CAD I and the Disrupt CAD II reported device times of 8 and 7.9 min, respectively. The Disrupt CAD II reported an IVL inflation time of 84.0 ± 59.7 s. The number of catheters was 1.2–2 (Disrupt CAD IV did not report). The number of pulses in the four trials ranged from 68.8 in the Disrupt CAD III to 104 in the Disrupt CAD IV, and IVL pressure was 6 atm. The number of stents used in IVL was about 1. The proportion of dilation has changed greatly: pre-dilation ranged from 20.3% in the Disrupt CAD IV and from 55.2% in the Disrupt CAD III, and post-dilation ranged from 1.6% in the Disrupt CAD IV and from 99.0% in the Disrupt CAD III (Table 4).

**Table 4:** Procedural details.

	**Disrupt CAD I**	**Disrupt CAD II**	**Disrupt CAD III**	**Disrupt CAD IV**	**Total**
Access type	Femoral	Femoral or radial	Femoral, radial, brachial, or ulnar	Femoral, radial, or brachial	
Total procedure time (min)	92 (70–109)	68.3 ± 34.2	53.0 (38.0–74.0)	62.5 ± 23.1	61.7 ± 30.2
Fluoroscopy time (min)	27 (18–41)	18.0 ± 11.1	15.0 (11.0–24.0)	22.2 ± 11.1	18.7 ± 11.6
Contrast volume (ml)	NA	181.9 ± 66.4	167.9 ± 71.9	NA	171.2 ± 70.8 [504]
Device time (min)	12 (8–17)	7.9 ± 5.2	NA	NA	9.4 ± 6.1 [180]
IVL inflation time (s)	NA	84.0 ± 59.7	NA	NA	84.0 ± 59.7 [120]
No. of catheters	2 (1,2)	1.2 ± 0.6	1.2 ± 0.5[377]	NA	1.2 ± 0.6 [557]
No. of pulses	72 (40,120)	70.7 ± 43.4	68.8 ± 31.9[377]	104.0 ± 56.0	73.6 ± 42.0 [621]
IVL pressure (atm)	6 (6,6)	5.8 ± 0.7	6.0 ± 0.3[377]	6.0 ± 0.0	6.0 ± 0.4 [621]
No. of stents used	1 (1,2)	1.3 ± 0.6	1.0 (1.0, 2.0)	1.1 ± 0.3	1.3 ± 0.7
Pre-dilation	22 (36.7%)	50 (41.7%)	212 (55.2%)	13 (20.3%)	297 (47.3%)
Post-dilation	52 (86.7%)	95 (79.2%)	377 (99.0%)[381]	1 (1.6%)	525 (84.0%) [625]

*Values are n (%) [N], or median with interquartile range (25%−75%), or mean ± standard deviation. NA, not applicable*.

### Safety Endpoint

Different trials reported MACE at different times. The Disrupt CAD I reported MACE through 30 days and 6 months and found that there were three (5.0%) patients who had non-Q-wave MI at 30 days and that two (3.3%) patients had cardiac death and three (5.0%) patients had non-Q-wave MI at 6 months. The Disrupt CAD II, Disrupt CAD III, and Disrupt CAD IV reported MACE in hospital and at 30 days. In the Disrupt CAD II, there were seven non-Q-wave MI in hospital and 10 MACE (one cardiac death, one Q-wave MI, seven non-Q-wave MI, and one TVR) at 30 days. In the Disrupt CAD III, there were 29 MACE (one cardiac death, four Q-wave MI, 22 non-Q-wave MI, and two TVR) in hospital and 37 MACE (two cardiac death, six Q-wave MI, 23 non-Q-wave MI, and six TVR) at 30 days. In the Disrupt CAD IV, there were four MACE (four non-Q-wave MI) in hospital and four MACE (four non-Q-wave MI) at 30 days (Table 5).

**Table 5:** Clinical outcomes.

	**Disrupt CAD I**	**Disrupt CAD II**	**Disrupt CAD III**	**Disrupt CAD IV**	**Total**
**Efficacy evaluation**
Procedural success	57 (95.0%)	113 (94.2%)	354 (92.2%)	60 (93.8%)	584 (93.0%)
Angiographic success	59 (98.3%)	120 (100.0%)	370 (96.4%)	63 (98.4%)	612 (97.5%)
Stent delivery	60 (100.0%)	120 (100.0%)	NA	NA	180 (100.0%) [180]
**MACE in hospital**
Cardiac death	NA	0 (0%)	1 (0.3%)	0 (0%)	1 (0.2%) [568]
Q-wave MI	NA	0 (0%)	4 (1.0%)	0 (0%)	4 (0.7%) [568]
Non-Q-wave MI	NA	7 (5.8%)	22 (5.7%)	4 (6.3%)	33 (5.8%) [568]
TVR	NA	0 (0%)	2 (0.5%)	0 (0%)	2 (0.4%) [568]
**MACE through 30 days**
Cardiac death	0 (0%)	1 (0.8%) [119]	2 (0.5%) [383]	0 (0%)	3 (0.5%) [568]
Q-wave MI	0 (0%)	1 (0.8%) [119]	6 (1.6%) [383]	0 (0%)	7 (1.2%) [568]
Non-Q-wave MI	3 (5.0%)	7 (5.9%) [119]	23 (6.0%) [383]	4 (6.3%)	37 (6.5%) [568]
TVR	0 (0%)	1 (0.8%) [119]	6 (1.6%) [383]	0 (0%)	7 (1.2%) [568]
**MACE through 6 months**
Cardiac death	2 (3.3%)	NA	NA	NA	2 (3.3%) [60]
Q-wave MI	0 (0%)	NA	NA	NA	0 (0%) [60]
Non-Q-wave MI	3 (5.0%)	NA	NA	NA	3 (5.0%) [60]
TVR	0 (0%)	NA	NA	NA	0 (0%) [60]

*Values are n (%) [N], or median with interquartile range (25%−75%), or mean ± standard deviation. MACE, major adverse cardiac event; MI, myocardial infarction; TVR, target vessel revascularization; NA, not applicable*.

### Efficacy Endpoint

IVL showed good efficacy. The Disrupt CAD I, Disrupt CAD II, Disrupt CAD III, and Disrupt CAD IV achieved 95.0, 94.2, 92.2, and 93.8% procedural success, respectively. Moreover, the Disrupt CAD I, Disrupt CAD II, Disrupt CAD III, and Disrupt CAD IV achieved 98.3, 100, 96.4, and 98.4% angiographic success, respectively (Table 5). Angiographic outcomes included final in-segment angiographic outcomes, final in-stent angiographic outcomes, and final angiographic complications. The Disrupt CAD I did not report final in-segment angiographic outcomes. In the other three trials, the minimum lumen diameter ranged from 2.42 to 2.83 mm, acute gain ranged from 1.41 to 1.63 mm, and residual diameter stenosis ranged from 9.4% to 17.8% (more than 98.4% of patients obtained residual diameter stenosis <50 and 94.8% of patients obtained residual diameter stenosis <30%). All trials reported final in-stent angiographic outcomes. The minimum lumen diameter ranged from 2.60 to 2.88 mm, acute gain ranged from 1.67 to 1.70 mm, and residual diameter stenosis ranged from 7.8 to 12.0% (all patients obtained residual diameter stenosis <50% and more than 91.7% of patients obtained residual diameter stenosis <30%). The Disrupt CAD I and Disrupt CAD IV reported no final angiographic complications, whereas the Disrupt CAD II reported two residual dissections (one type B and one type C, respectively). The Disrupt CAD III reported three final angiographic complications [one residual dissection (types D–F), one perforation, and one abrupt closure] (Table 6).

**Table 6:** Angiographic outcomes.

	**Disrupt CAD I**	**Disrupt CAD II**	**Disrupt CAD III**	**Disrupt CAD IV**	**Total**
**Final in-segment angiographic outcomes**
Minimum lumen diameter (mm)	NA	2.83 ± 0.48	2.47 ± 0.45	2.42 ± 0.40	2.5 ± 0.5 [568]
Acute gain, mm	NA	1.63 ± 0.49	1.41 ± 0.48	1.42 ± 0.42	1.5 ± 0.5 [568]
Residual diameter stenosis (%)	NA	9.4 ± 7.5	17.8 ± 8.8	15.9 ± 7.9	15.8 ± 9.1 [568]
Residual diameter stenosis <50%	NA	120 (100.0%)	381 (99.5%) [383]	63 (98.4%)	564 (99.5%) [567]
Residual diameter stenosis <30%	NA	119 (99.2%)	363 (94.8%) [383]	63 (98.4%)	545 (96.1%) [567]
**Final in-stent angiographic outcomes**
Minimum lumen diameter (mm)	2.6 (2.3–2.9)	2.88 ± 0.47	2.74 ± 0.43	2.67 ± 0.36	2.7 ± 0.4
Acute gain (mm)	1.7 (1.3–2.1)	1.67 ± 0.49	1.68 ± 0.46	1.67 ± 0.37	1.7 ± 0.5
Residual diameter stenosis (%)	12 (7–21)	7.8 ± 7.1	11.9 ± 7.1	9.9 ± 5.7	11.1 ± 7.6
Residual diameter stenosis <50%	60 (100.0%)	120 (100.0%)	381 (100.0%) [381]	64 (100.0%)	625 (100.0%) [625]
Residual diameter stenosis <30%	55 (91.7%)	120 (100.0%)	379 (99.5%) [381]	64 (100.0%)	585 (93.6%) [625]
Residual diameter stenosis <20%	44 (73.3%)	NA	NA	NA	44 (73.3%) [60]
**Final angiographic complications**
Residual dissections	0 (0.0%)	2 (1.7%)	1 (0.3%)	0 (0.0%)	3 (0.5%)
Perforations	0 (0.0%)	0 (0.0%)	1 (0.3%)	0 (0.0%)	1 (0.2%)
Abrupt closure	0 (0.0%)	0 (0.0%)	1 (0.3%)	0 (0.0%)	1 (0.2%)
Slow flow	0 (0.0%)	0 (0.0%)	0 (0.0%)	0 (0.0%)	0 (0.0%)
No reflow	0 (0.0%)	0 (0.0%)	0 (0.0%)	0 (0.0%)	0 (0.0%)

*Values are n (%) [N], or median with interquartile range (25%−75%), or mean ± standard deviation. NA, not applicable*.

### OCT Measurements

The Disrupt CAD II, Disrupt CAD III, and Disrupt CAD IV had OCT subgroup analysis.

Vessel preparation with IVL led to an increase in minimal luminal area from 2.33 ± 1.35 to 6.10 ± 2.17 mm^2^ in the Disrupt CAD II, from 2.16 ± 0.80 to 6.51 ± 2.03 mm^2^ in the Disrupt CAD III, and from 1.63 ± 0.69 to 5.85 ± 1.55 mm^2^ in the Disrupt CAD IV after drug-eluting stent (DES) implantation. The impact of IVL at the sites of the pre-IVL minimal luminal area, maximum calcium site, and final minimal stent area is shown in Table 7. IVL significantly increased the lumen area and decreased the calcium angle. Overall, calcium fracture was identified in 78.7% of lesions, with multiple fractures present in 55.3% in the Disrupt CAD II, in 70.4% of lesions with 51% multiple fractures in the Disrupt CAD III, and in 60.6% of lesions with 33.8% multiple fractures in the Disrupt CAD IV (Table 7). The maximum fracture depth and width and the maximum and minimum calcium angles at the fracture site are shown in Table 7.

**Table 7:** Serial OCT measurements and calcium fracture characteristics.

	**Disrupt CAD II**		**Disrupt CAD III**			**Disrupt CAD IV**			**Total**		
	**Pre-IVL (*N* = 48)**	**Post-stent (*N* = 47)**	**Pre-IVL (*N* = 97)**	**Post-IVL (*N* = 92)**	**Post-stent (*N* = 98)**	**Pre-IVL (*N* = 69)**	**Post-IVL (*N* = 71)**	**Post-stent (*N* = 71)**	**Pre-IVL (*N* = 214)**	**Post-IVL (*N* = 163)**	**Post-stent (*N* = 216)**
**At MLA site**											
Lumen area (mm^2^)	2.33 ± 1.35	6.10 ± 2.17	2.16 ± 0.80 [96]	3.57 ± 1.35 [92]	6.51 ± 2.03 [98]	1.63 ± 0.69 [69]	3.24 ± 1.36 [71]	5.85 ± 1.55 [71]	2.0 ± 1.0 [213]	3.4 ± 1.4 [163]	5.3 ± 2.4 [216]
Area stenosis	NA	NA	72.4 ± 11.6 [91]	56.1 ± 16.4 [84]	56.1 ± 16.4 [84]	74.5 ± 9.2 [62]	51.3 ± 16.4 [66]	13.5 ± 16.9 [67]	73.3 ± 10.7 [153]	54.0 ± 16.5 [150]	37.2 ± 26.9 [151]
Calcium angle (deg)	175.8 ± 96.9	127.1 ± 97.6 [28]	189.2 ± 96.0 [83]	151.2 ± 80.7 [67]	121.1 ± 71.1 [72]	152.1 ± 82.2 [53]	136.2 ± 76.4 [43]	129.9 ± 76.4 [53]	175.0 ± 93.3 [184]	145.3 ± 79.0 [110]	125.2 ± 77.9 [153]
Max. calcium thickness (mm)	0.9 ± 0.3	0.8 ± 0.3 [28]	0.87 ± 0.30 [83]	0.83 ± 0.28 [67]	0.83 ± 0.26 [72]	0.85 ± 0.30 [53]	0.84 ± 0.28 [43]	0.86 ± 0.25 [53]	0.9 ± 0.3 [184]	0.8 ± 0.3 [110]	0.8 ± 0.3 [153]
Stent area (mm^2^)		6.06 ± 2.20			6.53 ± 2.12 [98]			5.69 ± 1.44 [71]			6.2 ± 2.0 [216]
Stent expansion (%)		79.1 ± 21.0 [44]			78.2 ± 19.7 [94]			84.4 ± 16.6 [67]			80.4 ± 19.2 [205]
Acute area gain (mm^2^)		3.99 ± 1.72 [38]			NA			NA			3.99 ± 1.72 [38]
**At pre-IVL max. calcium site** ^**a**^											
Lumen area (mm^2^)	3.64 ± 1.78	8.47 ± 3.04 [38]	4.08 ± 2.32 [97]	5.86 ± 2.13 [91]	8.85 ± 2.23 [95]	3.65 ± 1.50 [69]	5.06 ± 1.49 [69]	7.38 ± 1.95 [69]	3.8 ± 2.0 [214]	5.5 ± 1.9 [160]	8.3 ± 2.4 [202]
Area stenosis	NA	NA	49.1 ± 28.0 [91]	26.6 ± 26.5 [83]	−8.2 ± 30.7 [91]	43.9 ± 30.5 [62]	20.8 ± 29.7 [64]	−9.3 ± 27.7 [65]	47.0 ± 29.1 [153]	24.1 ± 28.0 [147]	8.7 ± 29.2 [156]
Calcium angle (deg)	266.3 ± 77.1	215.1 ± 69.4 [38]	292.5 ± 76.5 [95]	257.5 ± 80.0 [91]	224.6 ± 75.0 [95]	257.9 ± 78.4 [69]	227.0 ± 80.0 [68]	209.5 ± 76.4 [69]	275.3 ± 78.5 [212]	244.5 ± 85.1 [159]	217.7 ± 74.4 [202]
Max. calcium thickness (mm)	0.93 ± 0.2	0.89 ± 0.2 [38]	0.96 ± 0.25 [95]	0.93 ± 0.21 [91]	0.89 ± 0.20 [95]	0.96 ± 0.27 [69]	0.92 ± 0.26 [68]	0.92 ± 0.26 [69]	1.0 ± 0.2 [212]	0.9 ± 0.2 [159]	0.9 ± 0.2 [202]
Stent area (mm^2^)		7.77 ± 2.65 [38]			8.30 ± 2.15 [94]			6.72 ± 1.82 [67]			7.7 ± 2.3 [199]
Stent expansion (%)		102.8 ± 30.6 [35]			101.7 ± 28.9 [90]			99.5 ± 23.5 [63]			101.2 ± 27.4 [188]
Acute area gain (mm^2^)		4.79 ± 2.45 [38]									4.79 ± 2.45 [38]
**At final MSA site**											
Lumen area (mm^2^)	4.26 ± 2.86	6.25 ± 2.25	4.15 ± 2.06 [89]	4.94 ± 1.94 [88]	6.66 ± 2.12 [98]	3.19 ± 1.83 [65]	4.10 ± 1.54 [71]	5.91 ± 1.57 [71]	3.9 ± 2.2 [202]	4.6 ± 1.8 [159]	6.3 ± 2.0 [216]
Area stenosis	NA	NA	47.8 ± 25.2 [84]	40.7 ± 22.9 [80]	20.0 ± 19.9 [94]	51.4 ± 24.1 [61]	36.6 ± 21.8 [66]	12.5 ± 17.5 [67]	49.3 ± 24.7 [145]	38.9 ± 22.5 [154]	16.9 ± 19.2 [161]
Calcium angle (deg)	176.6 ± 100.4 [23]	149.4 ± 94.8 [30]	157.0 ± 78.1 [66]	146.1 ± 76.8 [65]	128.9 ± 66.0 [71]	159.3 ± 88.5 [53]	145.2 ± 85.8 [57]	130.5 ± 78.0 [57]	161.0 ± 85.6 [142]	145.7 ± 80.8 [122]	133.4 ± 76.4 [158]
Max. calcium thickness (mm)	1.0 ± 0.3 [23]	0.9 ± 0.3 [30]	0.91 ± 0.24 [66]	0.88 ± 0.24 [65]	0.87 ± 0.24 [71]	0.89 ± 0.25 [53]	0.85 ± 0.26 [57]	0.84 ± 0.25 [57]	0.9 ± 0.3 [142]2	0.9 ± 0.2 [122]	0.9 ± 0.3 [158]
Stent area (mm^2^)		5.92 ± 2.14			6.47 ± 2.07 [98]			5.65 ± 1.45 [71]			6.1 ± 1.9 [216]
Stent expansion (%)		77.6 ± 20.5 [44]			78.4 ± 25.8 [94]			83.6 ± 16.1 [67]			80.0 ± 22.0 [205]
Acute area gain (mm^2^)		2.52 ± 2.03 [35]									2.52 ± 2.03 [35]
**Calcified nodule**	NA		18 (18.6%)			11 (15.9%)			29 (17.5%)		
**Calcium fracture analysis**											
Calcium fracture (%)		37 (78.7%)		62 (67.4%)	69 (70.4%)		38 (53.5%)	43 (60.6%)		100 (61.3%)	149 (69.0%)
1 fracture		11 (23.4%)		20 (21.7%)	19 (19.4%)		15 (21.1%)	19 (26.8%)		35 (21.5%)	49 (22.7%)
2 fractures		8 (17.0%)		15 (16.3%)	16 (16.3%)		5 (7.0%)	5 (7.0%)		20 (12.3%)	29 (13.4%)
≥3 fractures		18 (38.3%)		27 (29.3%)	34 (34.7%)		18 (25.4%)	19 (26.8%)		45 (27.6%)	71 (32.9%)
Max. fracture depth (mm)		0.6 ± 0.3 [37]		0.48 ± 0.25 [62]	0.49 ± 0.20 [69]		0.49 ± 0.23 [37]	0.51 ± 0.24 [43]		0.5 ± 0.2 [99]	0.5 ± 0.2 [149]
Max. fracture width (mm)		5.5 ± 5.0 [37]		0.55 ± 0.45 [62]	1.32 ± 1.04 [69]		0.59 ± 0.56 [37]	1.13 ± 0.95 [43]		0.6 ± 0.5 [99]	2.3 ± 3.2 [149]
Min. calcium angle at fracture site (deg)		224.5 ± 70.9 [37]		192.3 ± 67.0 [64]	173.5 ± 60.4 [69]		201.5 ± 73.2 [43]	182.9 ± 69.7 [43]		196.0 ± 69.4 [107]	188.9 ± 68.7 [149]
Max. calcium angle at fracture site (deg)		184.8 ± 64.8 [37]		263.7 ± 72.6 [64]	240.4 ± 73.2 [69]		243.5 ± 81.7 [43]	223.9 ± 82.1 [43]		255.6 ± 76.7 [107]	221.8 ± 76.8 [149]

*Values are n (%) [N] or mean ± standard deviation. MLA, minimal lumen area; IVL, intravascular lithotripsy; MSA, minimal stent area; NA, not applicable.*

a *Max calcium site was defined as the site with maximum calcium arc: if multiple sites had the same arc, the site with both maximum arc and thickness was selected*.

### Comparison With RA

We included ROTAXUS and PREPARE-CALC to pool the RA group, and the details of ROTAXUS and PREPARE-CALC are shown in Supplementary Table 1. We found more diabetes (38.4 vs. 30.0%), hyperlipidemia (84.6 vs. 72.3%), prior coronary artery bypass graft (9.6 vs. 6.8%), and patients with renal insufficiency (21.0 vs. 14.1%) in the Disrupt CAD group compared with the RA group (Supplementary Table 2). As for lesion characteristics, the target vessel involved more circumflex artery and right coronary artery and fewer protected left main artery and left anterior descending artery in the Disrupt CAD group than in the RA group (Supplementary Table 2). Moreover, since there were more severe calcification cases in the Disrupt CAD group (98.9 vs. 59.7%), the diameter stenosis was smaller (64.6 vs. 82.2%) than that in the RA group (Supplementary Table 2). Accordingly, the Disrupt CAD group had a shorter total procedure time (61.7 vs. 76.3 min), shorter fluoroscopy time (18.7 vs. 23.3 min), and smaller contrast volume (171.2 vs. 215.5 ml) than did the RA group (Supplementary Table 2). One case (0.2%) reported death and 37 cases (6.5%) reported MI among the 568 cases in the Disrupt CAD group, whereas two cases (0.9%) reported death and four cases (1.8%) reported MI among the 220 cases in the RA group. The procedural success, angiographic success, and stent delivery were 93.0, 97.5, and 100.0% in the Disrupt CAD group, and 95.0, 96.7, and 99.5% in the RA group, respectively (Supplementary Table 2). Angiographic outcomes are shown in Supplementary Table 2.

## Discussion

The serial Disrupt CAD trials evaluated the utility of IVL for lesion preparation of severely calcified coronary stenoses prior to stent implantation. Several major findings were derived from the study results. Firstly, both the primary safety and efficacy endpoints were met among patients from different regions, including the USA, Europe, and Japan. Secondly, coronary IVL prior to stent implantation was well tolerated, with a low rate of major periprocedural clinical and angiographic complications. Thirdly, the rate of 30-day MACE was low. Moreover, IVL achieved acute luminal gains and residual stenosis. Finally, OCT imaging provided evidence that calcium fracture was the underlying mechanism of action for coronary IVL.

Coronary revascularization is common. Researchers analyzed the preprocedural, in-hospital, and long-term data from the Coronary Revascularization Demonstrating Outcome Registry (Kyoto, Japan) and the Texas Heart Institute Research Database (Houston, Texas) of 16,100 patients who had undergone elective, initial percutaneous coronary intervention or coronary artery bypass grafting to compare the differences in the clinical characteristics and long-term outcomes of patients in these two countries. They found that the two registries showed similar crude outcomes, but for important differences in patient risk factors such as obesity, in the adjusted analysis, the Japanese patients had better outcomes than did the USA patients (31). The findings in this study including the serial Disrupt CAD trials, which cover a wide range of crowds, suggest that, despite underlying ethnic differences in the risk factors and the differing prevalences and morphologies of coronary artery plaques, the clinical outcomes of vessel preparation using IVL prior to stent placement are consistent among ethnic groups. In fact, in addition to the serial Disrupt CAD trials, other studies are exploring the safety and applicability of IVL in severely calcified coronary stenoses, but most of them are case reports and experience reports (32–37), so we did not include these low-quality studies, which are bound to have some impact on our conclusions.

IVL offers a novel option for severely calcified coronary stenoses. It is unique among all technologies due to its ability to modify calcium circumferentially and transmurally, which is provided by a diffuse acoustic pulse delivered through a low-pressure balloon as opposed to other devices that induce mechanical tissue injury, thus modifying transmural conduit compliance (2). The resulting potential clinical benefits of IVL include uniform plaque modification in which the fractured calcium remains *in situ* with no microcirculation embolization, thereby safely facilitating stent apposition and expansion (38). Previous long-term follow-up studies, such as ORBIT II (Evaluate the Safety and Efficacy of OAS in Treating Severely Calcified Coronary Lesions) (39) and COAST (Coronary Orbital Atherectomy System Study) (40), have confirmed that the incidence of MACE increases with the extension of the follow-up time. Here, we highlight its best clinical application through appropriate patient and lesion selection, with the main objective of optimizing stent delivery and implantation and, subsequently, improved outcomes. In view of the design of the serial Disrupt CAD trials, except for the Disrupt CAD I, the follow-up time was 6 months; the follow-up time of other studies was 30 days. In other words, we can only evaluate the immediate and short-term clinical utility, but not the long-term benefits of IVL. Secondly, IVL obviates the need for more complex lesion preparation strategies such as RA, except in severe undeletable cases where IVL is impossible (33). The serial Disrupt CAD trials are of a single-arm design, so we could not obtain data on the comparison between or the combination of IVL and RA or conventional balloon angioplasty (cutting or drug-coated balloons). Therefore, we introduced ROTAXUS and PREPARE-CALC to critically describe the basic information of these trials and the applicability and safety of IVL and RA. The previous retrospective study confirmed the high rate of procedural success and the low incidence of target lesion revascularization and MACE of RA in the European population (41), which are consistent with what we report here. Unfortunately, nearly one-third of the patients enrolled in ROTAXUS experienced MACE within a 2-year follow-up, with no differences between the patients treated with or without RA (42). However, because of the design and the statistical methods of these trials, we cannot directly compare IVL and RA. It is urgent and warranted to design high-quality trials to further directly compare the safety and efficacy between IVL and other methods. Thirdly, previous results have shown that radial access could reduce the hemorrhagic events and mortality compared to transfemoral access (43). In the Disrupt CAD I, PCI was all performed *via* femoral access; only femoral access was obtained in the Disrupt CAD II, whereas both femoral and radial access were obtained in the Disrupt CAD III and Disrupt CAD IV. Because the outcome indicators of the different approaches cannot be obtained alone, we regret that we cannot verify the superiority of the radial artery approach in prognosis. But it should be noted that femoral access is feasible in emergency, complications, or inability to use the radial access (43). Moreover, pivotal trials in acute coronary syndromes over the past several years have led to the introduction of novel antiplatelet agents, (44) such as prasugrel (45) and ticagrelor (46). The impact of these new agents on the complications of IVL is unknown and merits study. Finally, so far, no study has reported on the cost-effectiveness of IVL, which is necessary to be considered before clinical application.

## Conclusions

Ultimately, IVL is an efficient vessel preparation strategy in the presence of a heavy coronary calcium burden, and these results appear to be consistent regardless of ethnicity or geography. Moreover, calcium fracture facilitated increased vessel compliance and a favorable stent expansion. In addition, the impact of this technology on the long-term prognosis of patients with severe calcification is also the focus of attention and expectation. More importantly, the advantage of IVL over the other methods in this particular population is still unknown. Enhancing the comparison of IVL would help guide the therapeutic decisions in these patients. We hope that one day this technology can eventually replace the other coronary calcification treatment technologies currently used in clinical practice. By then, we will have a safe, efficient, and simple treatment method to treat severe calcification lesions accurately, rapidly, and efficiently.

## Data Availability Statement

The original contributions presented in the study are included in the article/Supplementary Material, further inquiries can be directed to the corresponding author/s.

## Ethics Statement

Ethical review and approval was not required for the study on human participants in accordance with the local legislation and institutional requirements. Written informed consent for participation was not required for this study in accordance with the national legislation and the institutional requirements.

## Author Contributions

BL and NG conceived, designed, or planned the idea. All authors acquired, analyzed and interpreted the data. BL drafted the manuscript. NG revised the manuscript. All authors read and approved the final manuscript.

## Conflict of Interest

The authors declare that the research was conducted in the absence of any commercial or financial relationships that could be construed as a potential conflict of interest.

## Publisher's Note

All claims expressed in this article are solely those of the authors and do not necessarily represent those of their affiliated organizations, or those of the publisher, the editors and the reviewers. Any product that may be evaluated in this article, or claim that may be made by its manufacturer, is not guaranteed or endorsed by the publisher.

## Abbreviations

CAC: coronary artery calcification
CAD: coronary artery disease
CK-MB: creatine kinase MB isoform
COAST: Coronary Orbital Atherectomy System Study
CTCA: computed tomography coronary angiography
IVUS: intravascular ultrasound
IVL: intravascular lithotripsy
MACE: major adverse cardiovascular events
MI: myocardial infarction
OCT: optical coherence tomography
ORBIT II: Evaluate the Safety and Efficacy of OAS in Treating Severely Calcified Coronary Lesions
PCI: percutaneous coronary intervention
PREPARE-CALC: Comparison of Strategies to Prepare Severely Calcified Coronary Lesions
RA: rotational atherectomy
ROTAXUS: Rotational Atherectomy Prior to TAXUS Stent Treatment for Complex Native Coronary Artery Disease
TVR: target vessel revascularization.
