# Supplementary material for: Evaluation of the Safety and Efficacy of Coronary Intravascular Lithotripsy for Treatment of Severely Calcified Coronary Stenoses: Evidence From the Serial Disrupt CAD Trials
Source: Front Cardiovasc Med. 2021 Aug 19;8:724481. doi: 10.3389/fcvm.2021.724481 (PMC8416910; doi:10.3389/fcvm.2021.724481)
Supplement: Supplementary file 1 [file Table_1.pdf]

Table S1. Summary of the RA group

	ROTAXUS	PREPARE-CALC
NCT Number	NCT00380809	NCT02502851
Design	Randomized active-controlled superiority trial performed at 3 high-volume, experienced interventional study sites in Germany	Randomized controlled trial performed at 2 high-volume, experienced interventional study sites in Germany
Inclusion criteria	Clinical Inclusion Criteria: 1. Age above 18 years, 2. Angiographically proven coronary artery disease, 3. Angina II to IV following the Canadian Cardiovascular Society classification criteria and/or reproducible ischemia in the target area by electrocardiogram or scintigraphy, 4. The patient signing an informed written consent.<br>Angiographic Inclusion Criteria*: First-degree criteria: 1. De-novo lesion in a native coronary artery, 2. Target reference vessel diameter between 2.5 and 4.0 mm by visual estimation, 3. Luminal diameter reduction of 70% to 99% by visual estimation, 4. Moderate to severe calcification of the target lesion**; Second-degree criteria: 1. Ostial location, 2. Bifurcational lesions, 3. Long lesions (≥15 mm),	Clinical Inclusion Criteria: 1. Age above 18 years and consentable, 2. Angiographically proven coronary artery disease, 3. Anginal symptoms and/or reproducible ischemia in the target area by electrocardiogram, functional stress testing or fractional flow reserve, 4. The patient signing an informed written consent.<br>Angiographic Inclusion Criteria: 1. De-novo lesion in a native coronary artery, 2. Target reference vessel diameter between 2.25 and 4.0 mm by visual estimation, 3. Luminal diameter reduction of 50~100% by visual estimation, 4. Severe calcification of the target lesion**
Exclusion criteria	Clinical Exclusion Criteria: 1. Myocardial infarction within 4 weeks, 2. Left ventricular ejection fraction < 30%, 3. Limited long-term prognosis due to other conditions.<br>Angiographic Exclusion Criteria: 1. Unprotected left main lesions, 2. Coronary artery bypass graft stenoses, 3. In-stent restenosis, 4. Chronic total occlusions, 5. Target vessel thrombus, 6. Target vessel dissection.	Clinical Exclusion Criteria: 1. Myocardial infarction within 1 week, 2. Decompensated heart failure, 3. Limited long term prognosis due to other conditions.<br>Angiographic exclusion criteria 1. Target lesion is in a coronary artery bypass graft, 2. Target lesion is an in-stent restenosis, 3. Target vessel thrombus.

*Lesions had to fulfill all first-degree criteria and at least 1 second-degree criterion to be eligible for inclusion. **Coronary calcium was angiographically graded as follows: none/mild; moderate (radiopacities noted only during the cardiac cycle before contrast injection); and severe (radiopacities noted without cardiac motion before contrast injection generally compromising both sides of the arterial lumen).
