# Supplementary material for: Evaluation of the Safety and Efficacy of Coronary Intravascular Lithotripsy for Treatment of Severely Calcified Coronary Stenoses: Evidence From the Serial Disrupt CAD Trials
Source: Front Cardiovasc Med. 2021 Aug 19;8:724481. doi: 10.3389/fcvm.2021.724481 (PMC8416910; doi:10.3389/fcvm.2021.724481)
Supplement: Supplementary file 2 [file Table_2.pdf]

Table S2. Comparison of Disrupt CAD and RA

	Disrupt CAD (N=628)	ROTAXUS (N=120)	PREPARE-CALC (N=100)	RA(N=220)
Baseline and clinical demographics				
Demographics				
Age	71.8±9.0	70.5±8.2	74.8±7.1	72.5±8.0
Male	484(77.1%)	86(71.7%)	77(77.0%)	163(74.1%)
Medical/surgical history				
Diabetes	241(38.4%)	33(27.5%)	33(33.0%)	66(30.0%)
Hypertension	539(85.8%)	106(88.3%)	93(93.0%)	199(90.5%)
Hyperlipidemia	531(84.6%)	91(75.8%)	68(68.0%)	159(72.3%)
Prior myocardial infarction	137(21.8%)	38(31.7%)	21(21.0%)	59(26.8%)
Prior coronary artery bypass graft	60(9.6%)	9(7.5%)	6(6.0%)	15(6.8%)
History of tobacco use	112(17.8%)	24(20.0%)	15(15.0%)	39(17.7%)
Renal insufficiency*	132(21.0%)	5(4.2%)	26(26.0%)	31(14.1%)
Lesion characteristics				
Target vessel				
Protected left main artery	9(1.4%)	3(2.1%)[146]	15(10.6%)[141]	18(6.3%)[287]
Left anterior descending artery	368(58.6%)	101(69.2%)[146]	78(55.3%)[141]	179(62.4%)[287]
Circumflex artery	75(11.9%)	7(4.8%)[146]	16(11.3%)[141]	23(8.0%)[287]
Right coronary artery	176(28%)	35(24.0%)[146]	32(22.7%)[141]	67(23.3%)[287]
Reference vessel diameter, mm	3.0±0.5	3.1±0.4[146]	3.25±0.47[141]	3.2±0.4[287]
Diameter stenosis, %	64.6±11.6	81.5±10.2[146]	83.02±10.35[141]	82.2±10.3[287]
Lesion length, mm	24.2±11.4	20.6±9.3[146]	29.81±15.23[141]	25.1±13.4[287]
Severe calcification	621(98.9%)	65(44.5%)[146]	104(75.9%)[137]	169(59.7%)[283]
Procedural details				

Total procedure time, min	61.7±30.2	66.4±44.5	88.2±34.9	76.3±41.8
Fluoroscopy time, min	18.7±11.6	22.8±21.9	23.9±12.2	23.3±18.1
Contrast volume, mL	171.2±70.8[504]	201.0±113.6	233.0±109.1	215.5±112.5
Number of stents used	1.3±0.7	1.3±0.6[146]	1.52±0.63[141]	1.4±0.6[287]
Clinical outcomes				
Efficacy evaluation				
Procedural success	584(93.0%)	111(92.5%)	98(98.0%)	209(95.0%)
Angiographic success	612(97.5%)	116(96.7%)	NA	116(96.7%)[120]
Stent delivery	180(100.0%)[180]	120(100%)	99(99.0%)	219(99.5%)
MACE in hospital				
Death	1(0.2%)[568]	2(1.7%)	0(0.0%)	2(0.9%)
MI	37(6.5%)[568]	2(1.7%)	2(2.0%)	4(1.8%)
MACE through 6 months				
Cardiac death	2(3.3%)[60]	NA	NA	NA
Q-wave MI	0(0%)[60]	NA	NA	NA
Non-Q-wave MI	3(5.0%)[60]	NA	NA	NA
TVR	0(0%)[60]	NA	NA	NA
MACE through 9 months		NA	NA	NA
Death	NA	6(5.3%)[113]	2(2.0%)	8(3.7%)[213]
MI	NA	8(7.1%)[113]	2(2.0%)	10(4.7%)[213]
TLR	NA	14(12.4%)[113]	2(2.0%)	16(7.5%)[213]
TVR	NA	20(17.7%)[113]	3(3.0%)	23(10.8%)[213]
Angiographic outcomes				
Final in-segment angiographic outcomes				
Minimum lumen diameter, mm	2.5±0.5[568]	2.26±0.49[146]	2.62±0.67[137]	2.4±0.6[283]
Acute gain, mm	1.5±0.5[568]	1.24±0.54[146]	1.47±0.64[137]	1.4±0.6[283]

Residual diameter stenosis, %	15.8±9.1[568]	18.21±8.77[146]	17.58±7.31[137]	17.9±8.1[283]
Final in-stent angiographic outcomes				
Minimum lumen diameter, mm	2.7±0.4	2.57±0.38[146]	2.85±0.43[137]	2.7±0.4[283]
Acute gain, mm	1.7±0.5	1.56±0.43[146]	1.70±0.42[137]	1.6±0.4[283]
Residual diameter stenosis, %	11.1±7.6	10.79±5.61[146]	12.62±5.36[137]	11.7±5.6[283]
Final angiographic complications				
Perforations	1(0.2%)	2(1.7%)	4(4.0%)	6(2.7%)
No/slow flow	0(0.0%)	0(0.0%)	2(2.0%)	2(0.9%)

Values are n (%)[N], or median with interquartile range (25%, 75%) or mean ± standard deviation. * Renal insufficiency was defined as estimated glomerular filtration rate < 60 mL/min/1.73m^2.

MACE: major adverse cardiac event, MI: myocardial infarction, TVR: target vessel revascularization, TLR: target lesion revascularization, NA: not applicable.
